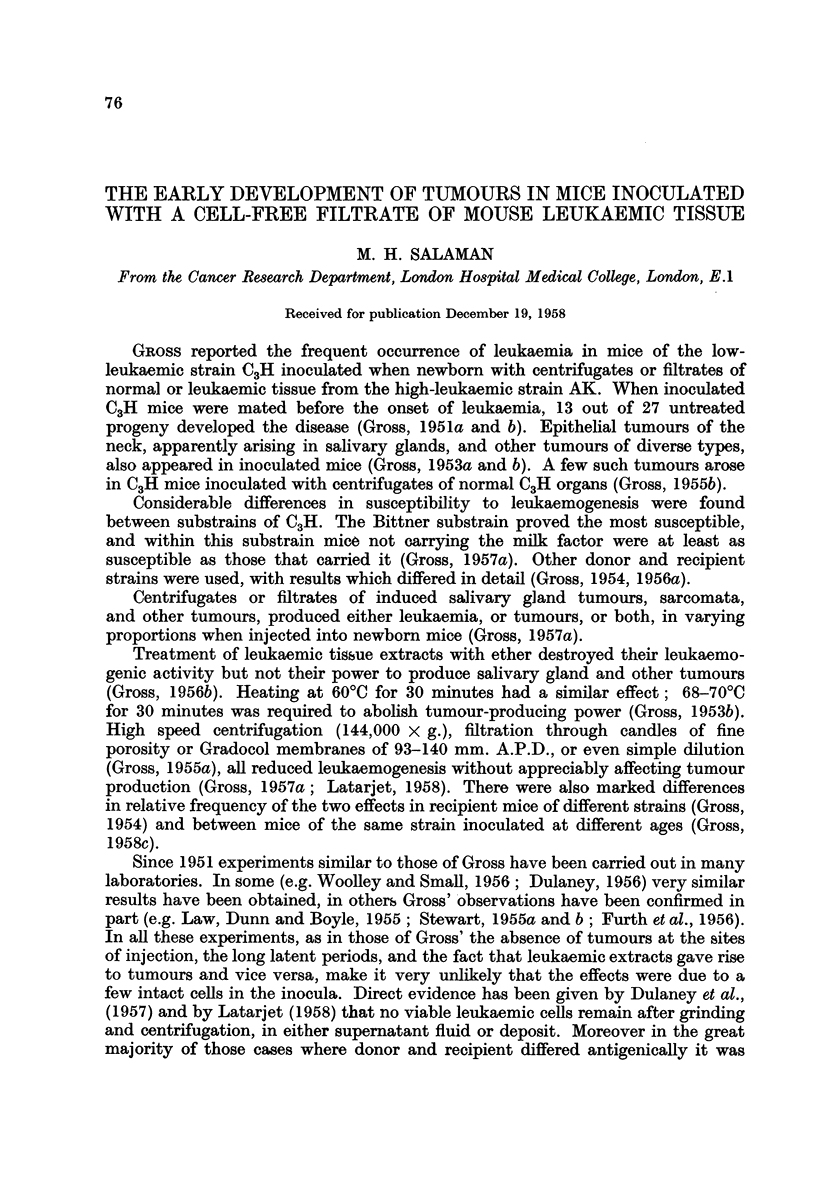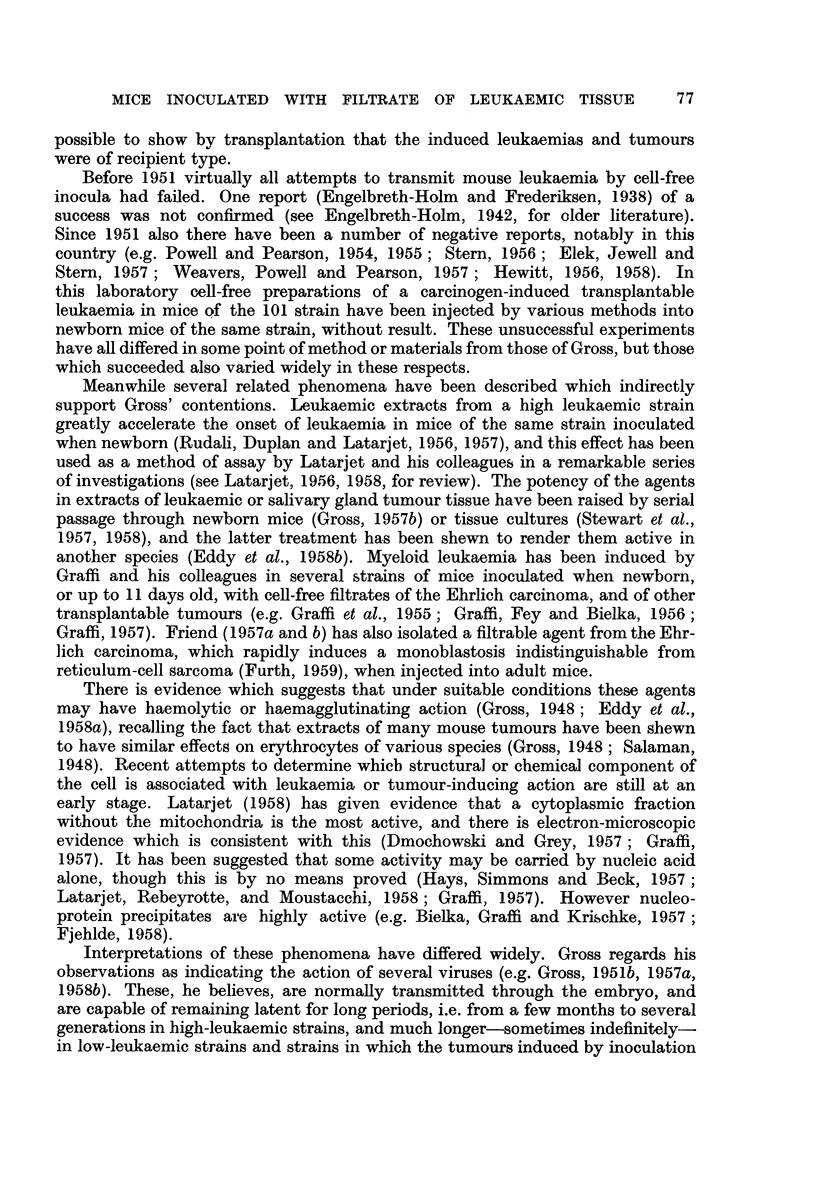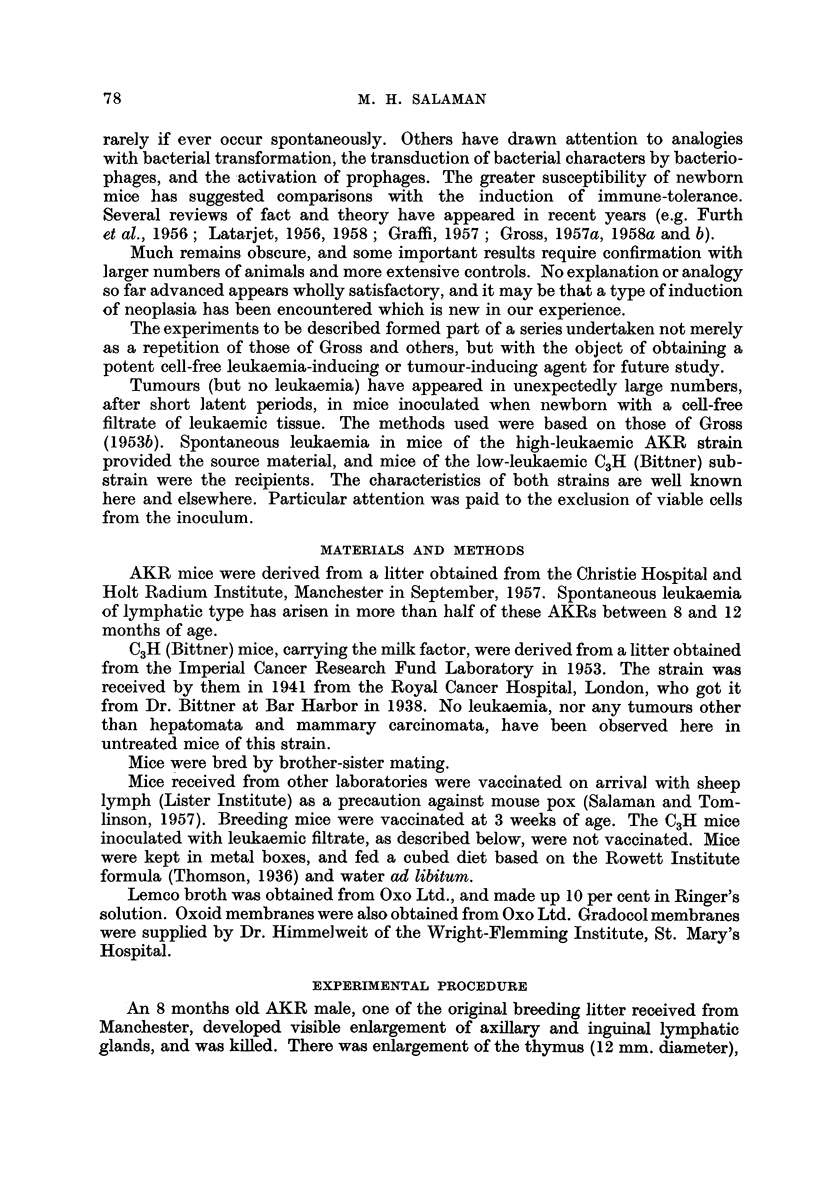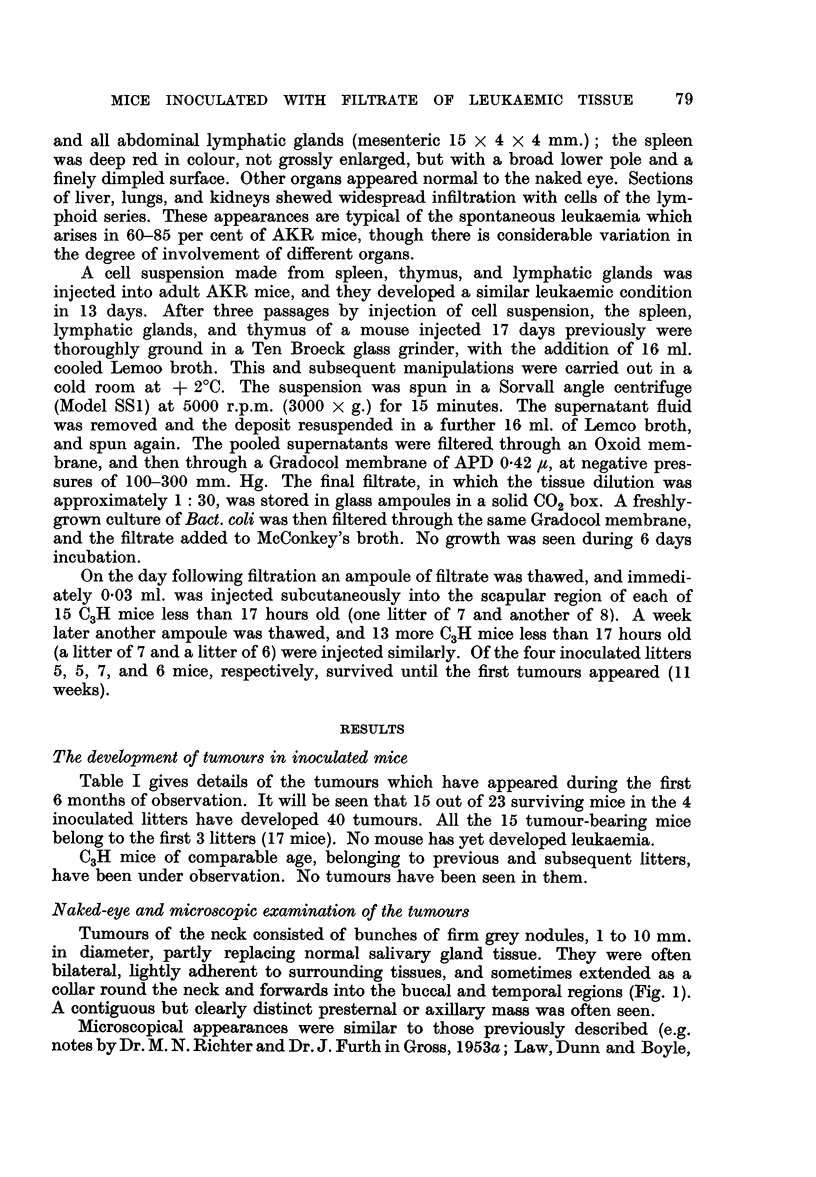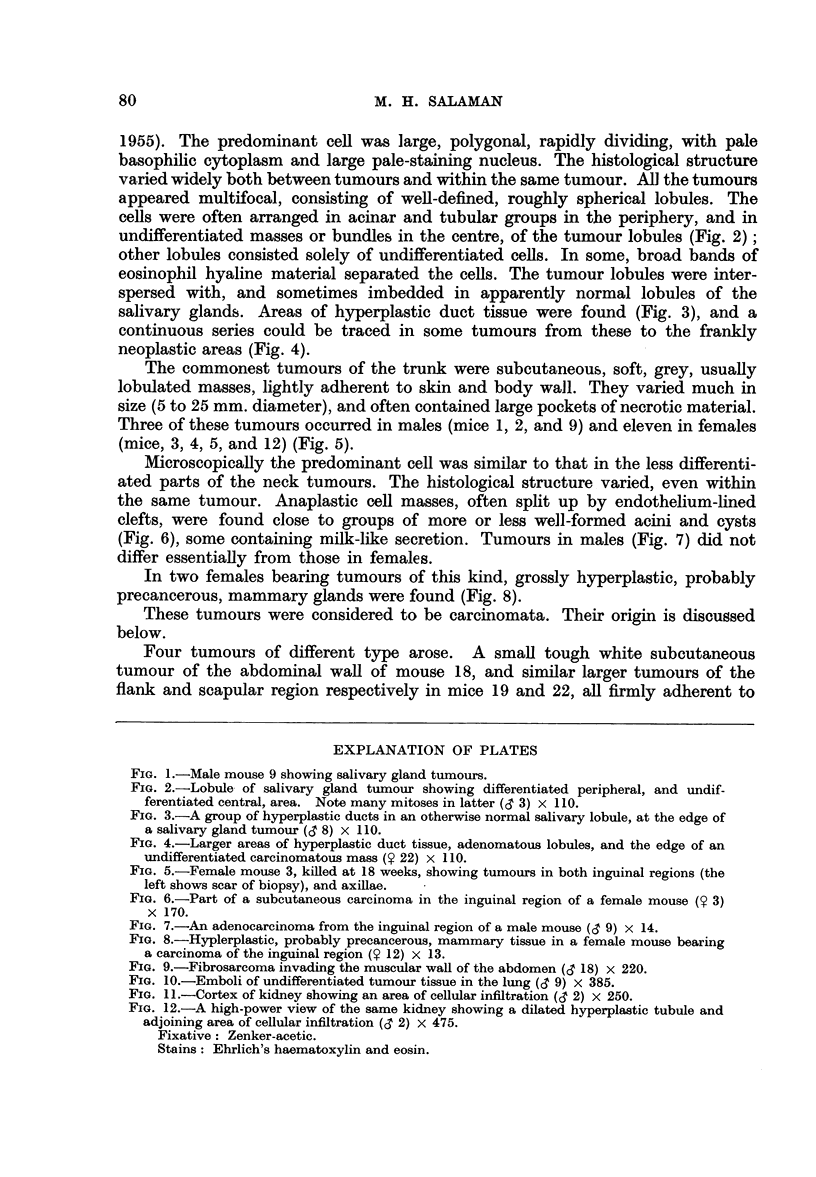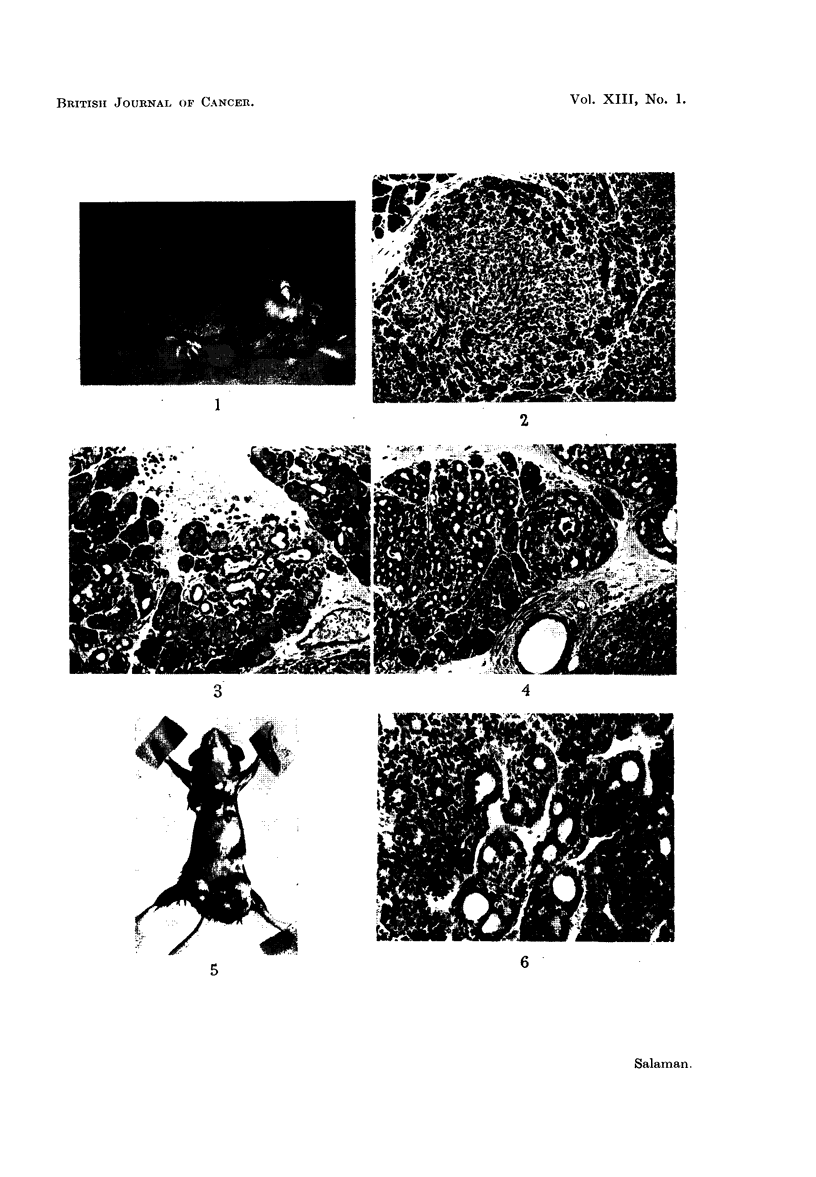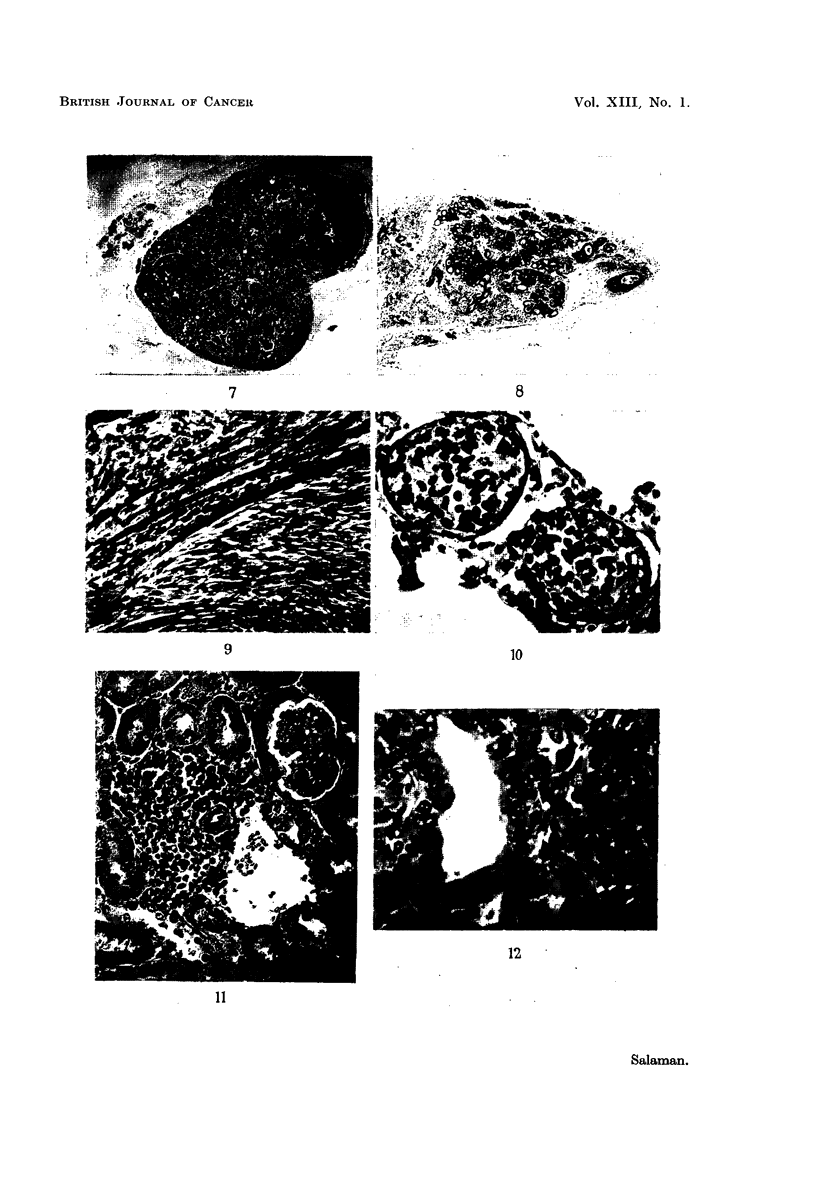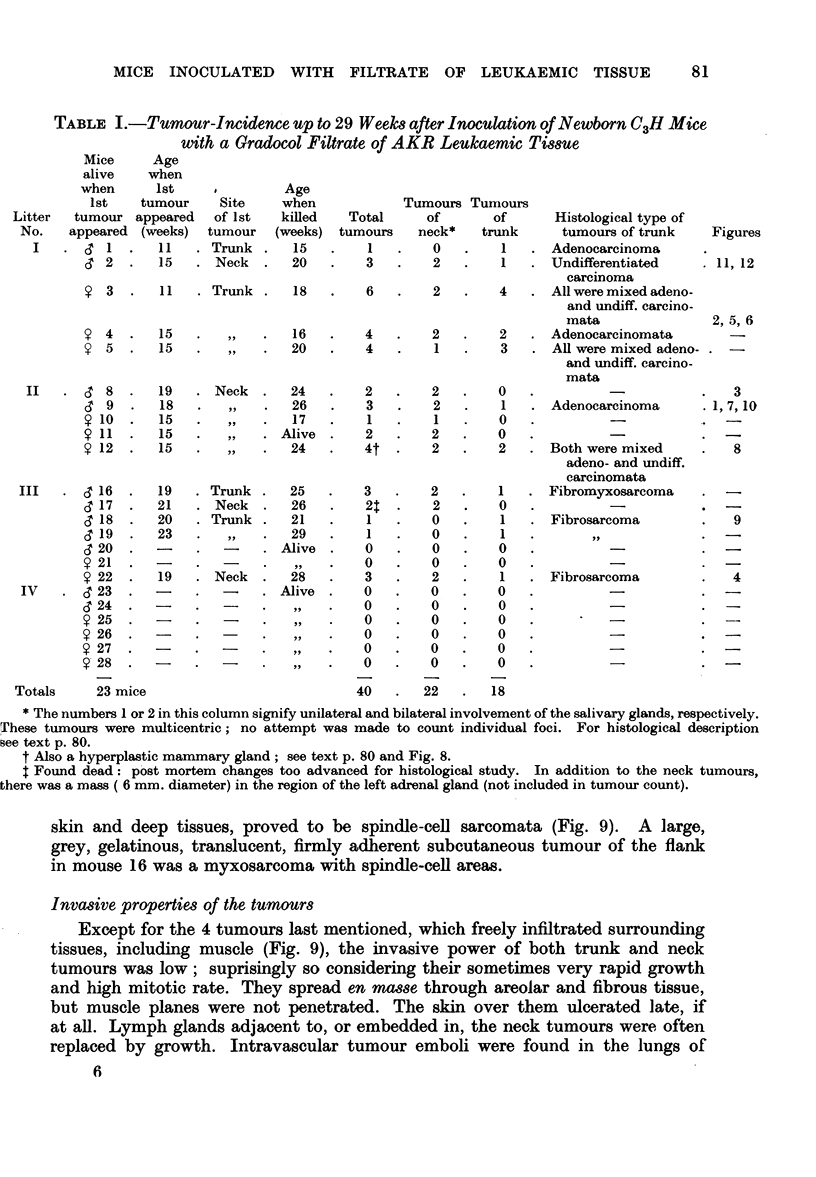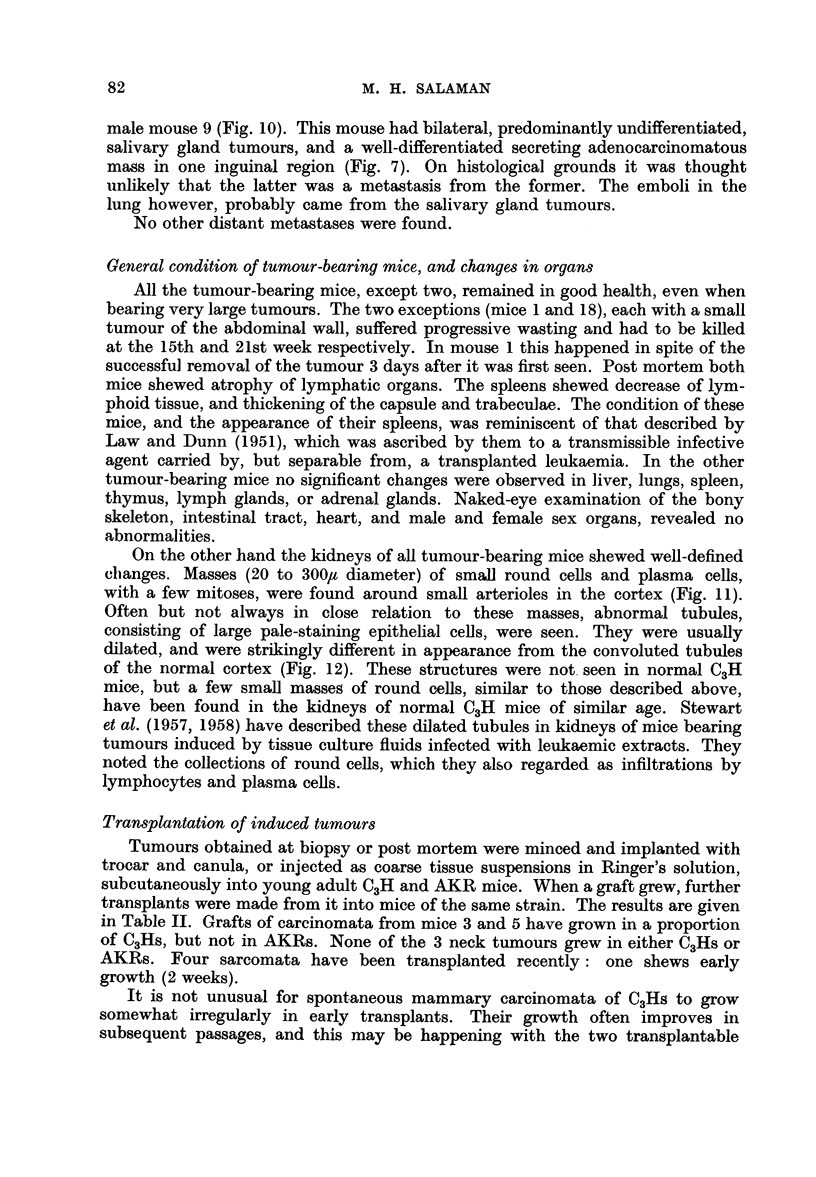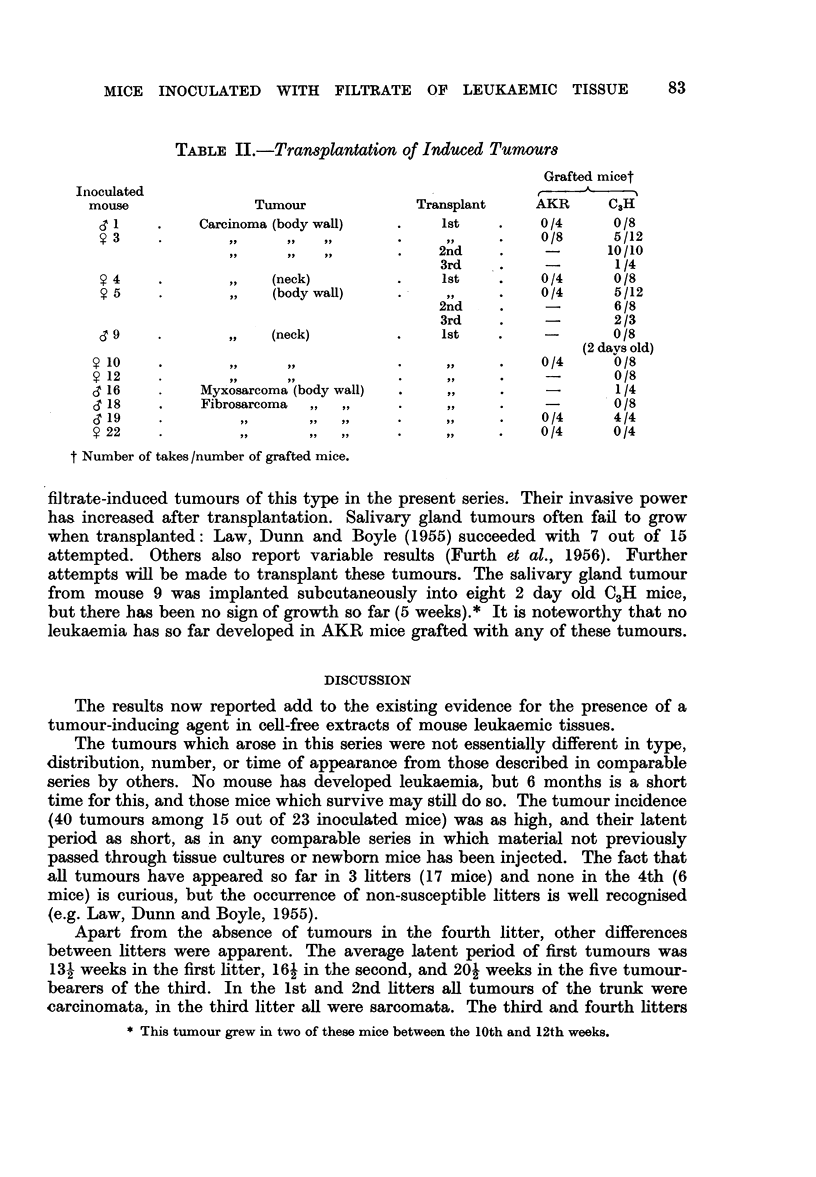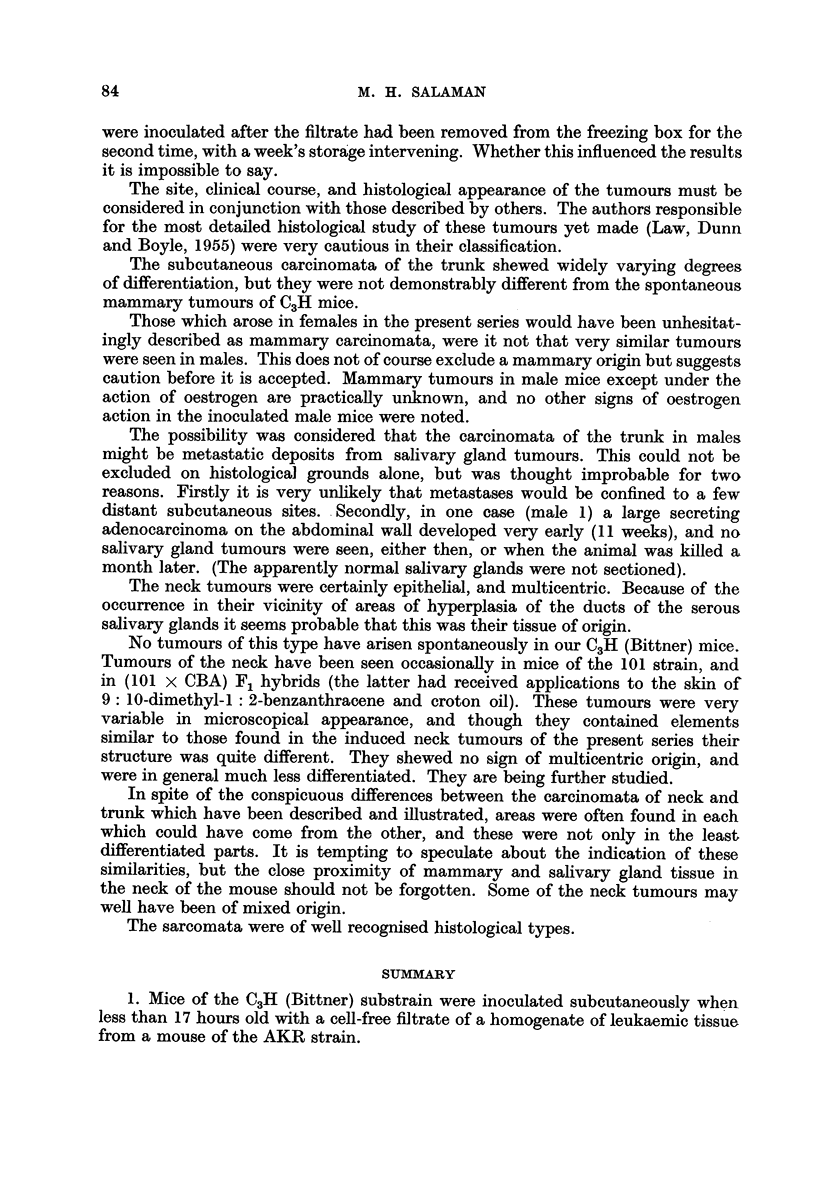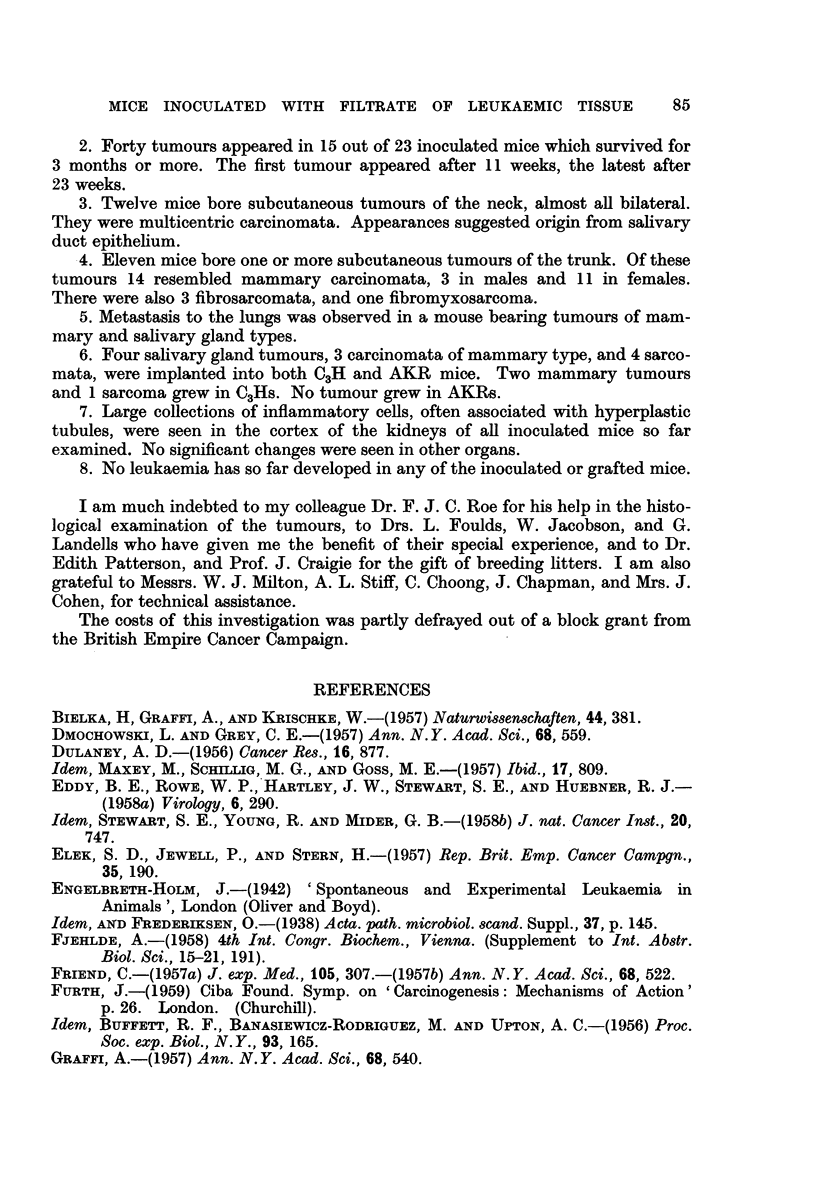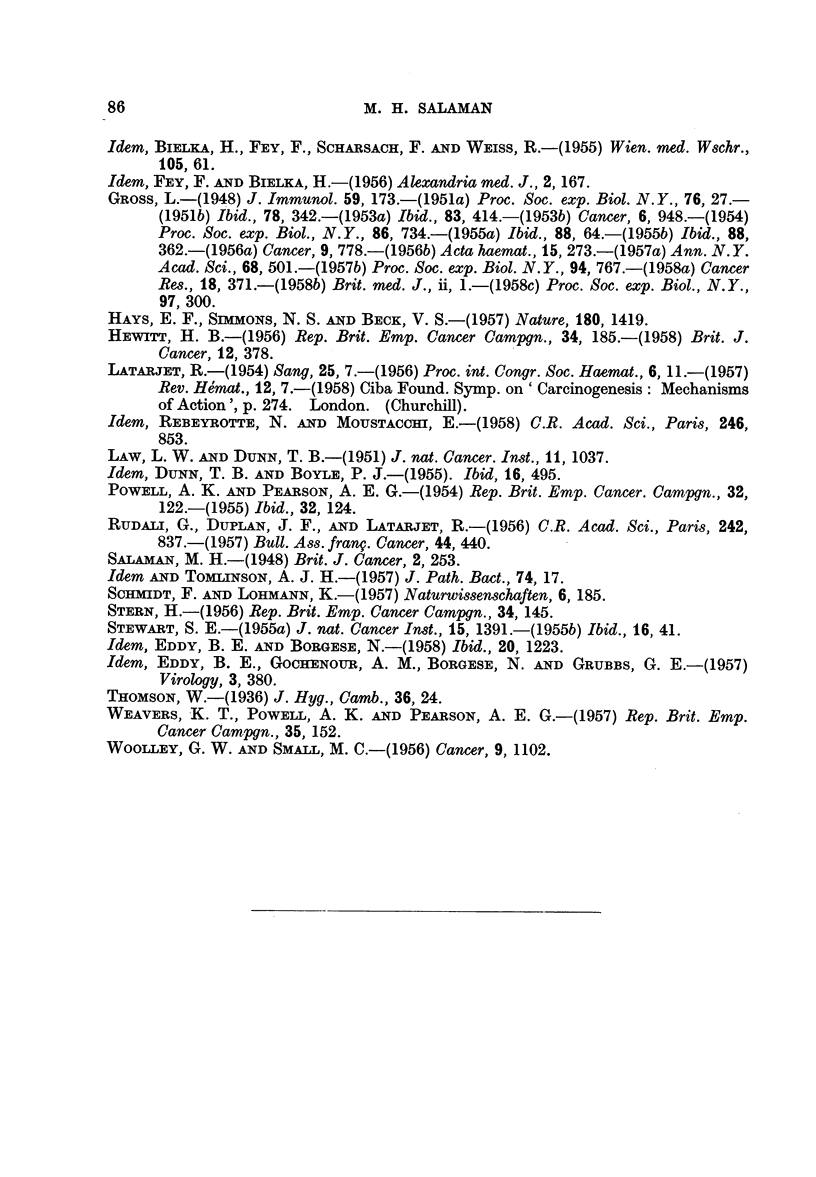# The Early Development of Tumours in Mice Inoculated with a Cell-Free Filtrate of Mouse Leukaemic Tissue

**DOI:** 10.1038/bjc.1959.10

**Published:** 1959-03

**Authors:** M. H. Salaman

## Abstract

**Images:**


### THE EARLY DEVELOPMENT OF TUMOURS IN MICE INOCULATED WITH A CELL-FREE FILTRATE OF MOUSE LEUKAEMIC TISSUE

M. H. SALAMAN

*From the Cancer Research Department, London Hospital Medical College, London, E.1*

Received for publication December 19, 1958

GROSS reported the frequent occurrence of leukaemia in mice of the low-leukaemic strain $C_3H$ inoculated when newborn with centrifugates or filtrates of normal or leukaemic tissue from the high-leukaemic strain AK. When inoculated $C_3H$ mice were mated before the onset of leukaemia, 13 out of 27 untreated progeny developed the disease (Gross, 1951*a* and *b*). Epithelial tumours of the neck, apparently arising in salivary glands, and other tumours of diverse types, also appeared in inoculated mice (Gross, 1953*a* and *b*). A few such tumours arose in $C_3H$ mice inoculated with centrifugates of normal $C_3H$ organs (Gross, 1955*b*).

Considerable differences in susceptibility to leukaemogenesis were found between substrains of $C_3H$. The Bittner substrain proved the most susceptible, and within this substrain mice not carrying the milk factor were at least as susceptible as those that carried it (Gross, 1957*a*). Other donor and recipient strains were used, with results which differed in detail (Gross, 1954, 1956*a*).

Centrifugates or filtrates of induced salivary gland tumours, sarcomata, and other tumours, produced either leukaemia, or tumours, or both, in varying proportions when injected into newborn mice (Gross, 1957*a*).

Treatment of leukaemic tissue extracts with ether destroyed their leukaemogenic activity but not their power to produce salivary gland and other tumours (Gross, 1956*b*). Heating at 60°C for 30 minutes had a similar effect ; 68–70°C for 30 minutes was required to abolish tumour-producing power (Gross, 1953*b*). High speed centrifugation (144,000 × g.), filtration through candles of fine porosity or Gradocol membranes of 93–140 mm. A.P.D., or even simple dilution (Gross, 1955*a*), all reduced leukaemogenesis without appreciably affecting tumour production (Gross, 1957*a* ; Latarjet, 1958). There were also marked differences in relative frequency of the two effects in recipient mice of different strains (Gross, 1954) and between mice of the same strain inoculated at different ages (Gross, 1958*c*).

Since 1951 experiments similar to those of Gross have been carried out in many laboratories. In some (e.g. Woolley and Small, 1956 ; Dulaney, 1956) very similar results have been obtained, in others Gross' observations have been confirmed in part (e.g. Law, Dunn and Boyle, 1955 ; Stewart, 1955*a* and *b* ; Furth *et al.*, 1956). In all these experiments, as in those of Gross' the absence of tumours at the sites of injection, the long latent periods, and the fact that leukaemic extracts gave rise to tumours and vice versa, make it very unlikely that the effects were due to a few intact cells in the inocula. Direct evidence has been given by Dulaney *et al.*, (1957) and by Latarjet (1958) that no viable leukaemic cells remain after grinding and centrifugation, in either supernatant fluid or deposit. Moreover in the great majority of those cases where donor and recipient differed antigenically it was possible to show by transplantation that the induced leukaemias and tumours were of recipient type.

Before 1951 virtually all attempts to transmit mouse leukaemia by cell-free inocula had failed. One report (Engelbreth-Holm and Frederiksen, 1938) of a success was not confirmed (see Engelbreth-Holm, 1942, for older literature). Since 1951 also there have been a number of negative reports, notably in this country (e.g. Powell and Pearson, 1954, 1955 ; Stern, 1956 ; Elek, Jewell and Stern, 1957 ; Weavers, Powell and Pearson, 1957 ; Hewitt, 1956, 1958). In this laboratory cell-free preparations of a carcinogen-induced transplantable leukaemia in mice of the 101 strain have been injected by various methods into newborn mice of the same strain, without result. These unsuccessful experiments have all differed in some point of method or materials from those of Gross, but those which succeeded also varied widely in these respects.

Meanwhile several related phenomena have been described which indirectly support Gross' contentions. Leukaemic extracts from a high leukaemic strain greatly accelerate the onset of leukaemia in mice of the same strain inoculated when newborn (Rudali, Duplan and Latarjet, 1956, 1957), and this effect has been used as a method of assay by Latarjet and his colleagues in a remarkable series of investigations (see Latarjet, 1956, 1958, for review). The potency of the agents in extracts of leukaemic or salivary gland tumour tissue have been raised by serial passage through newborn mice (Gross, 1957*b*) or tissue cultures (Stewart *et al.*, 1957, 1958), and the latter treatment has been shewn to render them active in another species (Eddy *et al.*, 1958*b*). Myeloid leukaemia has been induced by Graffi and his colleagues in several strains of mice inoculated when newborn, or up to 11 days old, with cell-free filtrates of the Ehrlich carcinoma, and of other transplantable tumours (e.g. Graffi *et al.*, 1955 ; Graffi, Fey and Bielka, 1956 ; Graffi, 1957). Friend (1957*a* and *b*) has also isolated a filtrable agent from the Ehrlich carcinoma, which rapidly induces a monoblastosis indistinguishable from reticulum-cell sarcoma (Furth, 1959), when injected into adult mice.

There is evidence which suggests that under suitable conditions these agents may have haemolytic or haemagglutinating action (Gross, 1948 ; Eddy *et al.*, 1958*a*), recalling the fact that extracts of many mouse tumours have been shewn to have similar effects on erythrocytes of various species (Gross, 1948 ; Salaman, 1948). Recent attempts to determine which structural or chemical component of the cell is associated with leukaemia or tumour-inducing action are still at an early stage. Latarjet (1958) has given evidence that a cytoplasmic fraction without the mitochondria is the most active, and there is electron-microscopic evidence which is consistent with this (Dmochowski and Grey, 1957 ; Graffi, 1957). It has been suggested that some activity may be carried by nucleic acid alone, though this is by no means proved (Hays, Simmons and Beck, 1957 ; Latarjet, Rebeyrotte, and Moustacchi, 1958 ; Graffi, 1957). However nucleoprotein precipitates are highly active (e.g. Bielka, Graffi and Krischke, 1957 ; Fjehlde, 1958).

Interpretations of these phenomena have differed widely. Gross regards his observations as indicating the action of several viruses (e.g. Gross, 1951*b*, 1957*a*, 1958*b*). These, he believes, are normally transmitted through the embryo, and are capable of remaining latent for long periods, i.e. from a few months to several generations in high-leukaemic strains, and much longer—sometimes indefinitely—in low-leukaemic strains and strains in which the tumours induced by inoculation rarely if ever occur spontaneously. Others have drawn attention to analogies with bacterial transformation, the transduction of bacterial characters by bacteriophages, and the activation of prophages. The greater susceptibility of newborn mice has suggested comparisons with the induction of immune-tolerance. Several reviews of fact and theory have appeared in recent years (e.g. Furth *et al.*, 1956 ; Latarjet, 1956, 1958 ; Graffi, 1957 ; Gross, 1957*a*, 1958*a* and *b*).

Much remains obscure, and some important results require confirmation with larger numbers of animals and more extensive controls. No explanation or analogy so far advanced appears wholly satisfactory, and it may be that a type of induction of neoplasia has been encountered which is new in our experience.

The experiments to be described formed part of a series undertaken not merely as a repetition of those of Gross and others, but with the object of obtaining a potent cell-free leukaemia-inducing or tumour-inducing agent for future study.

Tumours (but no leukaemia) have appeared in unexpectedly large numbers, after short latent periods, in mice inoculated when newborn with a cell-free filtrate of leukaemic tissue. The methods used were based on those of Gross (1953*b*). Spontaneous leukaemia in mice of the high-leukaemic AKR strain provided the source material, and mice of the low-leukaemic $C_3H$ (Bittner) substrain were the recipients. The characteristics of both strains are well known here and elsewhere. Particular attention was paid to the exclusion of viable cells from the inoculum.

### MATERIALS AND METHODS

AKR mice were derived from a litter obtained from the Christie Hospital and Holt Radium Institute, Manchester in September, 1957. Spontaneous leukaemia of lymphatic type has arisen in more than half of these AKRs between 8 and 12 months of age.

$C_3H$ (Bittner) mice, carrying the milk factor, were derived from a litter obtained from the Imperial Cancer Research Fund Laboratory in 1953. The strain was received by them in 1941 from the Royal Cancer Hospital, London, who got it from Dr. Bittner at Bar Harbor in 1938. No leukaemia, nor any tumours other than hepatomata and mammary carcinomata, have been observed here in untreated mice of this strain.

Mice were bred by brother-sister mating.

Mice received from other laboratories were vaccinated on arrival with sheep lymph (Lister Institute) as a precaution against mouse pox (Salaman and Tomlinson, 1957). Breeding mice were vaccinated at 3 weeks of age. The $C_3H$ mice inoculated with leukaemic filtrate, as described below, were not vaccinated. Mice were kept in metal boxes, and fed a cubed diet based on the Rowett Institute formula (Thomson, 1936) and water *ad libitum*.

Lemco broth was obtained from Oxo Ltd., and made up 10 per cent in Ringer's solution. Oxoid membranes were also obtained from Oxo Ltd. Gradocol membranes were supplied by Dr. Himmelweit of the Wright-Flemming Institute, St. Mary's Hospital.

### EXPERIMENTAL PROCEDURE

An 8 months old AKR male, one of the original breeding litter received from Manchester, developed visible enlargement of axillary and inguinal lymphatic glands, and was killed. There was enlargement of the thymus (12 mm. diameter), and all abdominal lymphatic glands (mesenteric 15 × 4 × 4 mm.); the spleen was deep red in colour, not grossly enlarged, but with a broad lower pole and a finely dimpled surface. Other organs appeared normal to the naked eye. Sections of liver, lungs, and kidneys shewed widespread infiltration with cells of the lymphoid series. These appearances are typical of the spontaneous leukaemia which arises in 60–85 per cent of AKR mice, though there is considerable variation in the degree of involvement of different organs.

A cell suspension made from spleen, thymus, and lymphatic glands was injected into adult AKR mice, and they developed a similar leukaemic condition in 13 days. After three passages by injection of cell suspension, the spleen, lymphatic glands, and thymus of a mouse injected 17 days previously were thoroughly ground in a Ten Broeck glass grinder, with the addition of 16 ml. cooled Lemco broth. This and subsequent manipulations were carried out in a cold room at + 2°C. The suspension was spun in a Sorvall angle centrifuge (Model SS1) at 5000 r.p.m. (3000 × g.) for 15 minutes. The supernatant fluid was removed and the deposit resuspended in a further 16 ml. of Lemco broth, and spun again. The pooled supernatants were filtered through an Oxoid membrane, and then through a Gradocol membrane of APD 0·42 $\mu$, at negative pressures of 100–300 mm. Hg. The final filtrate, in which the tissue dilution was approximately 1 : 30, was stored in glass ampoules in a solid $CO_2$ box. A freshly-grown culture of *Bact. coli* was then filtered through the same Gradocol membrane, and the filtrate added to McConkey's broth. No growth was seen during 6 days incubation.

On the day following filtration an ampoule of filtrate was thawed, and immediately 0·03 ml. was injected subcutaneously into the scapular region of each of 15 $C_3H$ mice less than 17 hours old (one litter of 7 and another of 8). A week later another ampoule was thawed, and 13 more $C_3H$ mice less than 17 hours old (a litter of 7 and a litter of 6) were injected similarly. Of the four inoculated litters 5, 5, 7, and 6 mice, respectively, survived until the first tumours appeared (11 weeks).

### RESULTS

### *The development of tumours in inoculated mice*

Table I gives details of the tumours which have appeared during the first 6 months of observation. It will be seen that 15 out of 23 surviving mice in the 4 inoculated litters have developed 40 tumours. All the 15 tumour-bearing mice belong to the first 3 litters (17 mice). No mouse has yet developed leukaemia.

$C_3H$ mice of comparable age, belonging to previous and subsequent litters, have been under observation. No tumours have been seen in them.

### *Naked-eye and microscopic examination of the tumours*

Tumours of the neck consisted of bunches of firm grey nodules, 1 to 10 mm. in diameter, partly replacing normal salivary gland tissue. They were often bilateral, lightly adherent to surrounding tissues, and sometimes extended as a collar round the neck and forwards into the buccal and temporal regions (Fig. 1). A contiguous but clearly distinct presternal or axillary mass was often seen.

Microscopical appearances were similar to those previously described (e.g. notes by Dr. M. N. Richter and Dr. J. Furth in Gross, 1953*a*; Law, Dunn and Boyle,

1955). The predominant cell was large, polygonal, rapidly dividing, with pale basophilic cytoplasm and large pale-staining nucleus. The histological structure varied widely both between tumours and within the same tumour. All the tumours appeared multifocal, consisting of well-defined, roughly spherical lobules. The cells were often arranged in acinar and tubular groups in the periphery, and in undifferentiated masses or bundles in the centre, of the tumour lobules (Fig. 2); other lobules consisted solely of undifferentiated cells. In some, broad bands of eosinophil hyaline material separated the cells. The tumour lobules were interspersed with, and sometimes imbedded in apparently normal lobules of the salivary glands. Areas of hyperplastic duct tissue were found (Fig. 3), and a continuous series could be traced in some tumours from these to the frankly neoplastic areas (Fig. 4).

The commonest tumours of the trunk were subcutaneous, soft, grey, usually lobulated masses, lightly adherent to skin and body wall. They varied much in size (5 to 25 mm. diameter), and often contained large pockets of necrotic material. Three of these tumours occurred in males (mice 1, 2, and 9) and eleven in females (mice, 3, 4, 5, and 12) (Fig. 5).

Microscopically the predominant cell was similar to that in the less differentiated parts of the neck tumours. The histological structure varied, even within the same tumour. Anaplastic cell masses, often split up by endothelium-lined clefts, were found close to groups of more or less well-formed acini and cysts (Fig. 6), some containing milk-like secretion. Tumours in males (Fig. 7) did not differ essentially from those in females.

In two females bearing tumours of this kind, grossly hyperplastic, probably precancerous, mammary glands were found (Fig. 8).

These tumours were considered to be carcinomata. Their origin is discussed below.

Four tumours of different type arose. A small tough white subcutaneous tumour of the abdominal wall of mouse 18, and similar larger tumours of the flank and scapular region respectively in mice 19 and 22, all firmly adherent to

---

EXPLANATION OF PLATES

FIG. 1.—Male mouse 9 showing salivary gland tumours.
FIG. 2.—Lobule of salivary gland tumour showing differentiated peripheral, and undifferentiated central, area. Note many mitoses in latter (♂ 3) × 110.
FIG. 3.—A group of hyperplastic ducts in an otherwise normal salivary lobule, at the edge of a salivary gland tumour (♂ 8) × 110.
FIG. 4.—Larger areas of hyperplastic duct tissue, adenomatous lobules, and the edge of an undifferentiated carcinomatous mass (♀ 22) × 110.
FIG. 5.—Female mouse 3, killed at 18 weeks, showing tumours in both inguinal regions (the left shows scar of biopsy), and axillae.
FIG. 6.—Part of a subcutaneous carcinoma in the inguinal region of a female mouse (♀ 3) × 170.
FIG. 7.—An adenocarcinoma from the inguinal region of a male mouse (♂ 9) × 14.
FIG. 8.—Hyplerplastic, probably precancerous, mammary tissue in a female mouse bearing a carcinoma of the inguinal region (♀ 12) × 13.
FIG. 9.—Fibrosarcoma invading the muscular wall of the abdomen (♂ 18) × 220.
FIG. 10.—Emboli of undifferentiated tumour tissue in the lung (♂ 9) × 385.
FIG. 11.—Cortex of kidney showing an area of cellular infiltration (♂ 2) × 250.
FIG. 12.—A high-power view of the same kidney showing a dilated hyperplastic tubule and adjoining area of cellular infiltration (♂ 2) × 475.

Fixative : Zenker-acetic.
Stains : Ehrlich's haematoxylin and eosin.

1

2

3

4

5

6

7

8

9

10

11

12

Table I.—*Tumour-Incidence up to 29 Weeks after Inoculation of Newborn $C_3H$ Mice with a Gradocol Filtrate of AKR Leukaemic Tissue*

| Litter No. | Mice alive when 1st tumour appeared | Age when 1st tumour appeared (weeks) | Site of 1st tumour | Age when killed (weeks) | Total tumours | Tumours of neck* | Tumours of trunk | Histological type of tumours of trunk | Figures |
|---|---|---|---|---|---|---|---|---|---|
| I | ♂ 1 | 11 | Trunk | 15 | 1 | 0 | 1 | Adenocarcinoma | |
| | ♂ 2 | 15 | Neck | 20 | 3 | 2 | 1 | Undifferentiated carcinoma | 11, 12 |
| | ♀ 3 | 11 | Trunk | 18 | 6 | 2 | 4 | All were mixed adeno- and undiff. carcinomata | 2, 5, 6 |
| | ♀ 4 | 15 | ,, | 16 | 4 | 2 | 2 | Adenocarcinomata | — |
| | ♀ 5 | 15 | ,, | 20 | 4 | 1 | 3 | All were mixed adeno- and undiff. carcinomata | — |
| II | ♂ 8 | 19 | Neck | 24 | 2 | 2 | 0 | — | 3 |
| | ♂ 9 | 18 | ,, | 26 | 3 | 2 | 1 | Adenocarcinoma | 1, 7, 10 |
| | ♀ 10 | 15 | ,, | 17 | 1 | 1 | 0 | — | — |
| | ♀ 11 | 15 | ,, | Alive | 2 | 2 | 0 | — | — |
| | ♀ 12 | 15 | ,, | 24 | 4† | 2 | 2 | Both were mixed adeno- and undiff. carcinomata | 8 |
| III | ♂ 16 | 19 | Trunk | 25 | 3 | 2 | 1 | Fibromyxosarcoma | — |
| | ♂ 17 | 21 | Neck | 26 | 2‡ | 2 | 0 | — | — |
| | ♂ 18 | 20 | Trunk | 21 | 1 | 0 | 1 | Fibrosarcoma | 9 |
| | ♂ 19 | 23 | ,, | 29 | 1 | 0 | 1 | ,, | — |
| | ♂ 20 | — | — | Alive | 0 | 0 | 0 | — | — |
| | ♀ 21 | — | — | ,, | 0 | 0 | 0 | — | — |
| | ♀ 22 | 19 | Neck | 28 | 3 | 2 | 1 | Fibrosarcoma | 4 |
| IV | ♂ 23 | — | — | Alive | 0 | 0 | 0 | — | — |
| | ♂ 24 | — | — | ,, | 0 | 0 | 0 | — | — |
| | ♀ 25 | — | — | ,, | 0 | 0 | 0 | — | — |
| | ♀ 26 | — | — | ,, | 0 | 0 | 0 | — | — |
| | ♀ 27 | — | — | ,, | 0 | 0 | 0 | — | — |
| | ♀ 28 | — | — | ,, | 0 | 0 | 0 | — | — |
| Totals | 23 mice | | | | 40 | 22 | 18 | | |

* The numbers 1 or 2 in this column signify unilateral and bilateral involvement of the salivary glands, respectively. These tumours were multicentric; no attempt was made to count individual foci. For histological description see text p. 80.

† Also a hyperplastic mammary gland; see text p. 80 and Fig. 8.

‡ Found dead: post mortem changes too advanced for histological study. In addition to the neck tumours, there was a mass ( 6 mm. diameter) in the region of the left adrenal gland (not included in tumour count).

skin and deep tissues, proved to be spindle-cell sarcomata (Fig. 9). A large, grey, gelatinous, translucent, firmly adherent subcutaneous tumour of the flank in mouse 16 was a myxosarcoma with spindle-cell areas.

*Invasive properties of the tumours*

Except for the 4 tumours last mentioned, which freely infiltrated surrounding tissues, including muscle (Fig. 9), the invasive power of both trunk and neck tumours was low; suprisingly so considering their sometimes very rapid growth and high mitotic rate. They spread *en masse* through areolar and fibrous tissue, but muscle planes were not penetrated. The skin over them ulcerated late, if at all. Lymph glands adjacent to, or embedded in, the neck tumours were often replaced by growth. Intravascular tumour emboli were found in the lungs of male mouse 9 (Fig. 10). This mouse had bilateral, predominantly undifferentiated, salivary gland tumours, and a well-differentiated secreting adenocarcinomatous mass in one inguinal region (Fig. 7). On histological grounds it was thought unlikely that the latter was a metastasis from the former. The emboli in the lung however, probably came from the salivary gland tumours.

No other distant metastases were found.

*General condition of tumour-bearing mice, and changes in organs*

All the tumour-bearing mice, except two, remained in good health, even when bearing very large tumours. The two exceptions (mice 1 and 18), each with a small tumour of the abdominal wall, suffered progressive wasting and had to be killed at the 15th and 21st week respectively. In mouse 1 this happened in spite of the successful removal of the tumour 3 days after it was first seen. Post mortem both mice shewed atrophy of lymphatic organs. The spleens shewed decrease of lymphoid tissue, and thickening of the capsule and trabeculae. The condition of these mice, and the appearance of their spleens, was reminiscent of that described by Law and Dunn (1951), which was ascribed by them to a transmissible infective agent carried by, but separable from, a transplanted leukaemia. In the other tumour-bearing mice no significant changes were observed in liver, lungs, spleen, thymus, lymph glands, or adrenal glands. Naked-eye examination of the bony skeleton, intestinal tract, heart, and male and female sex organs, revealed no abnormalities.

On the other hand the kidneys of all tumour-bearing mice shewed well-defined changes. Masses (20 to 300$\mu$ diameter) of small round cells and plasma cells, with a few mitoses, were found around small arterioles in the cortex (Fig. 11). Often but not always in close relation to these masses, abnormal tubules, consisting of large pale-staining epithelial cells, were seen. They were usually dilated, and were strikingly different in appearance from the convoluted tubules of the normal cortex (Fig. 12). These structures were not seen in normal $C_3H$ mice, but a few small masses of round cells, similar to those described above, have been found in the kidneys of normal $C_3H$ mice of similar age. Stewart *et al.* (1957, 1958) have described these dilated tubules in kidneys of mice bearing tumours induced by tissue culture fluids infected with leukaemic extracts. They noted the collections of round cells, which they also regarded as infiltrations by lymphocytes and plasma cells.

*Transplantation of induced tumours*

Tumours obtained at biopsy or post mortem were minced and implanted with trocar and canula, or injected as coarse tissue suspensions in Ringer's solution, subcutaneously into young adult $C_3H$ and AKR mice. When a graft grew, further transplants were made from it into mice of the same strain. The results are given in Table II. Grafts of carcinomata from mice 3 and 5 have grown in a proportion of $C_3Hs$, but not in AKRs. None of the 3 neck tumours grew in either $C_3Hs$ or AKRs. Four sarcomata have been transplanted recently: one shews early growth (2 weeks).

It is not unusual for spontaneous mammary carcinomata of $C_3Hs$ to grow somewhat irregularly in early transplants. Their growth often improves in subsequent passages, and this may be happening with the two transplantable

TABLE II.—*Transplantation of Induced Tumours*

| Inoculated mouse | Tumour | Transplant | Grafted mice† AKR | Grafted mice† $C_3H$ |
|---|---|---|---|---|
| ♂ 1 | Carcinoma (body wall) | 1st | 0/4 | 0/8 |
| ♀ 3 | „ „ „ | „ | 0/8 | 5/12 |
| | „ „ „ | 2nd | — | 10/10 |
| | | 3rd | — | 1/4 |
| ♀ 4 | „ (neck) | 1st | 0/4 | 0/8 |
| ♀ 5 | „ (body wall) | „ | 0/4 | 5/12 |
| | | 2nd | — | 6/8 |
| | | 3rd | — | 2/3 |
| ♂ 9 | „ (neck) | 1st | — | 0/8 (2 days old) |
| ♀ 10 | „ „ | „ | 0/4 | 0/8 |
| ♀ 12 | „ „ | „ | — | 0/8 |
| ♂ 16 | Myxosarcoma (body wall) | „ | — | 1/4 |
| ♂ 18 | Fibrosarcoma „ „ | „ | — | 0/8 |
| ♂ 19 | „ „ „ | „ | 0/4 | 4/4 |
| ♀ 22 | „ „ „ | „ | 0/4 | 0/4 |

† Number of takes/number of grafted mice.

filtrate-induced tumours of this type in the present series. Their invasive power has increased after transplantation. Salivary gland tumours often fail to grow when transplanted: Law, Dunn and Boyle (1955) succeeded with 7 out of 15 attempted. Others also report variable results (Furth *et al.*, 1956). Further attempts will be made to transplant these tumours. The salivary gland tumour from mouse 9 was implanted subcutaneously into eight 2 day old $C_3H$ mice, but there has been no sign of growth so far (5 weeks).* It is noteworthy that no leukaemia has so far developed in AKR mice grafted with any of these tumours.

### DISCUSSION

The results now reported add to the existing evidence for the presence of a tumour-inducing agent in cell-free extracts of mouse leukaemic tissues.

The tumours which arose in this series were not essentially different in type, distribution, number, or time of appearance from those described in comparable series by others. No mouse has developed leukaemia, but 6 months is a short time for this, and those mice which survive may still do so. The tumour incidence (40 tumours among 15 out of 23 inoculated mice) was as high, and their latent period as short, as in any comparable series in which material not previously passed through tissue cultures or newborn mice has been injected. The fact that all tumours have appeared so far in 3 litters (17 mice) and none in the 4th (6 mice) is curious, but the occurrence of non-susceptible litters is well recognised (e.g. Law, Dunn and Boyle, 1955).

Apart from the absence of tumours in the fourth litter, other differences between litters were apparent. The average latent period of first tumours was 13½ weeks in the first litter, 16½ in the second, and 20½ weeks in the five tumour-bearers of the third. In the 1st and 2nd litters all tumours of the trunk were carcinomata, in the third litter all were sarcomata. The third and fourth litters

* This tumour grew in two of these mice between the 10th and 12th weeks.

were inoculated after the filtrate had been removed from the freezing box for the second time, with a week's storage intervening. Whether this influenced the results it is impossible to say.

The site, clinical course, and histological appearance of the tumours must be considered in conjunction with those described by others. The authors responsible for the most detailed histological study of these tumours yet made (Law, Dunn and Boyle, 1955) were very cautious in their classification.

The subcutaneous carcinomata of the trunk shewed widely varying degrees of differentiation, but they were not demonstrably different from the spontaneous mammary tumours of $C_3H$ mice.

Those which arose in females in the present series would have been unhesitatingly described as mammary carcinomata, were it not that very similar tumours were seen in males. This does not of course exclude a mammary origin but suggests caution before it is accepted. Mammary tumours in male mice except under the action of oestrogen are practically unknown, and no other signs of oestrogen action in the inoculated male mice were noted.

The possibility was considered that the carcinomata of the trunk in males might be metastatic deposits from salivary gland tumours. This could not be excluded on histological grounds alone, but was thought improbable for two reasons. Firstly it is very unlikely that metastases would be confined to a few distant subcutaneous sites. Secondly, in one case (male 1) a large secreting adenocarcinoma on the abdominal wall developed very early (11 weeks), and no salivary gland tumours were seen, either then, or when the animal was killed a month later. (The apparently normal salivary glands were not sectioned).

The neck tumours were certainly epithelial, and multicentric. Because of the occurrence in their vicinity of areas of hyperplasia of the ducts of the serous salivary glands it seems probable that this was their tissue of origin.

No tumours of this type have arisen spontaneously in our $C_3H$ (Bittner) mice. Tumours of the neck have been seen occasionally in mice of the 101 strain, and in (101 × CBA) $F_1$ hybrids (the latter had received applications to the skin of 9 : 10-dimethyl-1 : 2-benzanthracene and croton oil). These tumours were very variable in microscopical appearance, and though they contained elements similar to those found in the induced neck tumours of the present series their structure was quite different. They shewed no sign of multicentric origin, and were in general much less differentiated. They are being further studied.

In spite of the conspicuous differences between the carcinomata of neck and trunk which have been described and illustrated, areas were often found in each which could have come from the other, and these were not only in the least differentiated parts. It is tempting to speculate about the indication of these similarities, but the close proximity of mammary and salivary gland tissue in the neck of the mouse should not be forgotten. Some of the neck tumours may well have been of mixed origin.

The sarcomata were of well recognised histological types.

### SUMMARY

1. Mice of the $C_3H$ (Bittner) substrain were inoculated subcutaneously when less than 17 hours old with a cell-free filtrate of a homogenate of leukaemic tissue from a mouse of the AKR strain.

2. Forty tumours appeared in 15 out of 23 inoculated mice which survived for 3 months or more. The first tumour appeared after 11 weeks, the latest after 23 weeks.

3. Twelve mice bore subcutaneous tumours of the neck, almost all bilateral. They were multicentric carcinomata. Appearances suggested origin from salivary duct epithelium.

4. Eleven mice bore one or more subcutaneous tumours of the trunk. Of these tumours 14 resembled mammary carcinomata, 3 in males and 11 in females. There were also 3 fibrosarcomata, and one fibromyxosarcoma.

5. Metastasis to the lungs was observed in a mouse bearing tumours of mammary and salivary gland types.

6. Four salivary gland tumours, 3 carcinomata of mammary type, and 4 sarcomata, were implanted into both $C_3H$ and AKR mice. Two mammary tumours and 1 sarcoma grew in $C_3Hs$. No tumour grew in AKRs.

7. Large collections of inflammatory cells, often associated with hyperplastic tubules, were seen in the cortex of the kidneys of all inoculated mice so far examined. No significant changes were seen in other organs.

8. No leukaemia has so far developed in any of the inoculated or grafted mice.

I am much indebted to my colleague Dr. F. J. C. Roe for his help in the histological examination of the tumours, to Drs. L. Foulds, W. Jacobson, and G. Landells who have given me the benefit of their special experience, and to Dr. Edith Patterson, and Prof. J. Craigie for the gift of breeding litters. I am also grateful to Messrs. W. J. Milton, A. L. Stiff, C. Choong, J. Chapman, and Mrs. J. Cohen, for technical assistance.

The costs of this investigation was partly defrayed out of a block grant from the British Empire Cancer Campaign.

### REFERENCES

Bielka, H, Graffi, A., and Krischke, W.—(1957) *Naturwissenschaften*, **44**, 381.

Dmochowski, L. and Grey, C. E.—(1957) *Ann. N.Y. Acad. Sci.*, **68**, 559.

Dulaney, A. D.—(1956) *Cancer Res.*, **16**, 877.

*Idem*, Maxey, M., Schillig, M. G., and Goss, M. E.—(1957) *Ibid.*, **17**, 809.

Eddy, B. E., Rowe, W. P., Hartley, J. W., Stewart, S. E., and Huebner, R. J.—(1958*a*) *Virology*, **6**, 290.

*Idem*, Stewart, S. E., Young, R. and Mider, G. B.—(1958*b*) *J. nat. Cancer Inst.*, **20**, 747.

Elek, S. D., Jewell, P., and Stern, H.—(1957) *Rep. Brit. Emp. Cancer Campgn.*, **35**, 190.

Engelbreth-Holm, J.—(1942) 'Spontaneous and Experimental Leukaemia in Animals', London (Oliver and Boyd).

*Idem*, and Frederiksen, O.—(1938) *Acta. path. microbiol. scand.* Suppl., **37**, p. 145.

Fjehlde, A.—(1958) *4th Int. Congr. Biochem., Vienna.* (Supplement to *Int. Abstr. Biol. Sci.*, 15–21, 191).

Friend, C.—(1957*a*) *J. exp. Med.*, **105**, 307.—(1957*b*) *Ann. N.Y. Acad. Sci.*, **68**, 522.

Furth, J.—(1959) Ciba Found. Symp. on 'Carcinogenesis: Mechanisms of Action' p. 26. London. (Churchill).

*Idem*, Buffett, R. F., Banasiewicz-Rodriguez, M. and Upton, A. C.—(1956) *Proc. Soc. exp. Biol., N.Y.*, **93**, 165.

Graffi, A.—(1957) *Ann. N.Y. Acad. Sci.*, **68**, 540.

*Idem*, BIELKA, H., FEY, F., SCHARSACH, F. AND WEISS, R.—(1955) *Wien. med. Wschr.*, **105**, 61.
*Idem*, FEY, F. AND BIELKA, H.—(1956) *Alexandria med. J.*, **2**, 167.
GROSS, L.—(1948) *J. Immunol.* **59**, 173.—(1951*a*) *Proc. Soc. exp. Biol. N.Y.*, **76**, 27.—(1951*b*) *Ibid.*, **78**, 342.—(1953*a*) *Ibid.*, **83**, 414.—(1953*b*) *Cancer*, **6**, 948.—(1954) *Proc. Soc. exp. Biol., N.Y.*, **86**, 734.—(1955*a*) *Ibid.*, **88**, 64.—(1955*b*) *Ibid.*, **88**, 362.—(1956*a*) *Cancer*, **9**, 778.—(1956*b*) *Acta haemat.*, **15**, 273.—(1957*a*) *Ann. N.Y. Acad. Sci.*, **68**, 501.—(1957*b*) *Proc. Soc. exp. Biol. N.Y.*, **94**, 767.—(1958*a*) *Cancer Res.*, **18**, 371.—(1958*b*) *Brit. med. J.*, ii, 1.—(1958*c*) *Proc. Soc. exp. Biol., N.Y.*, **97**, 300.
HAYS, E. F., SIMMONS, N. S. AND BECK, V. S.—(1957) *Nature*, **180**, 1419.
HEWITT, H. B.—(1956) *Rep. Brit. Emp. Cancer Campgn.*, **34**, 185.—(1958) *Brit. J. Cancer*, **12**, 378.
LATARJET, R.—(1954) *Sang*, **25**, 7.—(1956) *Proc. int. Congr. Soc. Haemat.*, **6**, 11.—(1957) *Rev. Hémat.*, **12**, 7.—(1958) Ciba Found. Symp. on ' Carcinogenesis : Mechanisms of Action ', p. 274. London. (Churchill).
*Idem*, REBEYROTTE, N. AND MOUSTACCHI, E.—(1958) *C.R. Acad. Sci., Paris*, **246**, 853.
LAW, L. W. AND DUNN, T. B.—(1951) *J. nat. Cancer. Inst.*, **11**, 1037.
*Idem*, DUNN, T. B. AND BOYLE, P. J.—(1955). *Ibid*, **16**, 495.
POWELL, A. K. AND PEARSON, A. E. G.—(1954) *Rep. Brit. Emp. Cancer. Campgn.*, **32**, 122.—(1955) *Ibid.*, **32**, 124.
RUDALI, G., DUPLAN, J. F., AND LATARJET, R.—(1956) *C.R. Acad. Sci., Paris*, **242**, 837.—(1957) *Bull. Ass. franç. Cancer*, **44**, 440.
SALAMAN, M. H.—(1948) *Brit. J. Cancer*, **2**, 253.
*Idem* AND TOMLINSON, A. J. H.—(1957) *J. Path. Bact.*, **74**, 17.
SCHMIDT, F. AND LOHMANN, K.—(1957) *Naturwissenschaften*, **6**, 185.
STERN, H.—(1956) *Rep. Brit. Emp. Cancer Campgn.*, **34**, 145.
STEWART, S. E.—(1955*a*) *J. nat. Cancer Inst.*, **15**, 1391.—(1955*b*) *Ibid.*, **16**, 41.
*Idem*, EDDY, B. E. AND BORGESE, N.—(1958) *Ibid.*, **20**, 1223.
*Idem*, EDDY, B. E., GOCHENOUR, A. M., BORGESE, N. AND GRUBBS, G. E.—(1957) *Virology*, **3**, 380.
THOMSON, W.—(1936) *J. Hyg., Camb.*, **36**, 24.
WEAVERS, K. T., POWELL, A. K. AND PEARSON, A. E. G.—(1957) *Rep. Brit. Emp. Cancer Campgn.*, **35**, 152.
WOOLLEY, G. W. AND SMALL, M. C.—(1956) *Cancer*, **9**, 1102.